# Genetic characterization of primary lateral sclerosis

**DOI:** 10.1007/s00415-023-11746-7

**Published:** 2023-05-03

**Authors:** Eva M. J. de Boer, Balint S. de Vries, Maartje Pennings, Erik-Jan Kamsteeg, Jan H. Veldink, Leonard H. van den Berg, Michael A. van Es

**Affiliations:** 1 Department of Neurology, Brain Center Rudolf Magnus, University Medical Center Utrecht, Utrecht, The Netherlands; 2 Department of Human Genetics, Radboud University Medical Center, Nijmegen, The Netherlands

**Keywords:** Primary lateral sclerosis, Amyotrophic lateral sclerosis, Hereditary spastic paraplegia, Genetics

## Abstract

**Background and objectives:**

Primary lateral sclerosis (PLS) is a motor neuron disease characterised by loss of the upper motor neurons. Most patients present with slowly progressive spasticity of the legs, which may also spread to the arms or bulbar regions. It is challenging to distinguish between PLS, early-stage amyotrophic lateral sclerosis (ALS) and hereditary spastic paraplegia (HSP). The current diagnostic criteria advise against extensive genetic testing. This recommendation is, however, based on limited data.

**Methods:**

We aim to genetically characterize a PLS cohort using whole exome sequencing (WES) for genes associated with ALS, HSP, ataxia and movement disorders (364 genes) and *C9orf72* repeat expansions. Patients fulfilling the definite PLS criteria by Turner et al. and with available DNA samples of sufficient quality were recruited from an on-going, population-based epidemiological study. Genetic variants were classified according to the ACMG criteria and assigned to groups based on disease association.

**Results:**

WES was performed in 139 patients and the presence of repeat expansions in *C9orf72* was analysed separately in 129 patients. This resulted in 31 variants of which 11 were (likely) pathogenic. (Likely) pathogenic variants resulted in 3 groups based on disease association: ALS-FTD (*C9orf72*, *TBK1*), pure HSP (*SPAST*, *SPG7*), “ALS-HSP-CMT overlap” (*FIG4*, *NEFL*, *SPG11*).

**Discussion:**

In a cohort of 139 PLS patients, genetic analyses resulted in 31 variants (22%) of which 10 (7%) (likely) pathogenic associated with different diseases (predominantly ALS and HSP). Based on these results and the literature, we advise to consider genetic analyses in the diagnostic work-up for PLS.

**Supplementary Information:**

The online version contains supplementary material available at 10.1007/s00415-023-11746-7.

## Introduction

Primary lateral sclerosis (PLS) is a rare neurodegenerative disorder characterized by the predominant loss of upper motor neurons (UMNs) [1]. It is considered to be one of the extremes of the motor neuron disease (MND) spectrum, which in turn forms a continuum with frontotemporal dementia (FTD) [2]. Indeed, studies show that approximately 50% of PLS patients may also develop cognitive and/or behavioural changes within the FTD-spectrum [3, 4].

The exact incidence and prevalence of PLS are unknown, but it is estimated that they make up roughly 1–5% of all MND patients seen at specialized clinics [1, 5]. Diagnosing PLS is challenging, over the years several diagnostic criteria have been implemented [6–8], but to date there is no gold standard (including post-mortem histopathological analysis). The diagnosis is based on recognizing characteristic clinical features and ruling out potential other causes of observed symptoms. Patients most commonly present with a history of slowly progressive spasticity in the legs which over time spreads cranially or more rarely with a spastic dysarthria that spreads caudally to cause spasticity in the extremities. Subtle signs of Parkinsonism have also been reported in PLS patients [1, 7, 9].

Through the use of ancillary investigations, such as MRI-scans and laboratory testing, several conditions with similar clinical presentations can be ruled out relatively straightforward [e.g., primary progressive multiple sclerosis and X-linked adrenoleukodystrophy (X-ALD)]. However, for other conditions in the differential diagnosis, such as amyotrophic lateral sclerosis (ALS) and hereditary spastic paraplegia (HSP) this is more complex. The initial onset of ALS may be characterized by UMN signs only with lower motor neuron loss occurring later in the disease course [2]. Therefore, follow-up over a number of years is frequently required to confidently establish the absence of lower motor neuron involvement, which is considered to be a defining feature of PLS.

HSP is a heterogeneous group of genetic disorders characterized by slowly progressive spasticity of the legs (pure forms) or with additional features such as epilepsy, learning and developmental problems and hearing loss (complex forms) [1, 10, 11]. In general, onset of HSP is at younger age than in PLS, ranging from childhood to early adulthood, symptoms are symmetrical and limited to the lower extremities, progression is slower and there commonly is a positive family history for the disease [10, 12]. However, asymptomatic UMN signs in the cervical region are frequently seen in HSP cases [13], late-onset in the late fifties and early sixties has also been reported, as well as asymmetry [14–16]. The mode of inheritance in HSP can also be recessive or X-linked, or the cause can be de novo mutations, therefore suggesting an apparently negative family history [16]. What complicates matters even further is the fact that to date over 80 genetic HSP subtypes have been identified, but which still do not explain all cases, and that therefore negative DNA test results do not rule out HSP [11]. Lastly, recent pathology studies have shown that PSP may also present with isolated UMN signs and therefore mimic PLS [9]. PLS is a clinical syndrome and may be considered as a collection of primary pyramidal disorders with heterogeneous causes, which might be subclassified more specifically using genetics.

As our knowledge of neurodegenerative diseases has advanced over the last decades, the diagnostic criteria for PLS have been revised a number of times to incorporate these novel insights. The current consensus criteria (2020) state the following regarding genetic testing “Screening of panels for pathogenic genetic variants associated with spastic paraparesis (e.g., *SPAST*) is warranted in cases of progressive UMN syndromes restricted to symmetrical lower limb involvement. It is reasonable to routinely exclude the most common hereditary cause of ALS in Caucasian populations, namely an expansion in *C9orf72.* However, the plethora of very rare genetic variants reported in association with pedigrees containing ALS-like syndromes, including some with apparently pure UMN phenotypes, should *not* be considered routine tests in the diagnosis of PLS”[8]. Although this statement reflects international consensus, it must be noted that only a limited number of very small studies have been conducted on the genetics of PLS and that the data supporting this statement is quite limited. We therefore set out to perform an in-depth genetic characterization of a large cohort of primary lateral sclerosis patients from The Netherlands using whole exome sequencing with the goal of providing additional guidance to clinicians with respect to genetic testing and/or to refine the diagnostic criteria for PLS.

## Methods

### Study population

All participants were recruited through an on-going population-based, epidemiological study on MND in The Netherlands (PAN study), which started in 2006 and was approved by the medical-ethical committee of the University Medical Center Utrecht. [17] Written informed consent was obtained from all participants. In this study, patients consent to the use of data from their medical records for scientific research, are asked to fill in detailed questionnaires and provide bio-samples, including DNA. We included patients of whom a DNA sample of sufficient quality was present and which fulfilled the most recent Turner criteria for definite PLS. These criteria require age at diagnosis to be ≥ 25 years, isolated UMN signs in at least two regions, and a disease duration of more than 4 years [8].

Baseline clinical data were collected at diagnosis or within a 3-month time window after diagnosis, and included: gender, age at diagnosis, disease duration (from onset and from diagnosis), site of disease onset, regions of UMN involvement (bulbar, cervical, thoracic, lumbosacral), cognitive and/or behavioural changes (by history taking and if available Edinburgh Cognitive and behavioural ALS screen, Frontal Assessment Battery and ALS and Frontotemporal Dementia Questionnaire[18–20]) and family history. In cases of only one region of UMN involvement at diagnosis, follow-up data (if available) was reviewed to see if patients developed UMN dysfunction in more regions making them fulfil the diagnostic criteria (cases remaining in one region or without available follow-up data were excluded). Site of disease onset was divided into: spinal, bulbar, or respiratory. Date of symptom onset was determined at the outpatient clinic as described in our Standard Operating Procedures (SOP) for Clinical Trials 2014.

The reporting of this study conforms to the STROBE statement [21].

### Genetic analyses

DNA samples were analysed at the department of human genetics of the Radboud university medical center using whole exome sequencing (WES). Capture of exons was done using an Agilent SureSelect Human All Exon 50 Mb Kit (Santa Clara, CA, USA) and WES was performed using Illumina HiSeq, both done by BGI-Europe (Copenhagen, Denmark). Median coverage depth was at least 80x. Subsequently, read mapping and variant calling were done using BWA (mapping) and GATK (calling) and copy number variant analyses were done using CoNIFER. Short tandem repeat analysis in the WES data was done using ExpansionHunter [22]. The datasets were analyzed using an in-house annotation pipeline and manual interpretation by a clinical laboratory geneticist [23]. In cases of detected short tandem repeats (STR’s) through WES, repeat-primed polymerase chain reaction (PCR’s) were performed as confirmation [24].

The presence of a *C9orf72* repeat expansion was determined separately through a repeat-primed PCR for the *C9orf72* hexanucleotide repeat the University Medical Centre Utrecht as described previously [25] Patients with more than 30 hexanucleotide repeats in the *C9orf72* gene were considered as *C9orf72* carriers [26] In case of repeat expansions additional PCR and fragment length analysis using GeneScan were performed [27].

### Gene panel

Considering that the main diagnostic challenge with regards to PLS is making the distinction with ALS, HSP and perhaps to a lesser extent atypical forms of parkinsonism, we analyzed a panel consisting of genes associated with MND-FTD and “movement disorders” including HSP, dystonia, Parkinsonism and ataxias. A total of 364 genes (338 genes see Appendix A, 22 genes Appendix B and *GRN*, *MAPT*, *NEK1* and *C21orf2*) were analysed using WES. Part of the movement disorders gene panel test is the analysis of STR’s involved in autosomal dominant spinocerebellar ataxia’s: SCA1 (*ATNX1*), SCA2 (*AXTN2*), SCA3 (*ATXN3*), SCA6 (*CACANA1A*), SCA7 (*ATXN7*), SCA10 (*ATXN10*), SCA12 (*PPP2R2B*), SCA17 (*TBP*), SCA36 (*NOP36*), Friedreich ataxia (*FXN*) and dentatorubral-pallidoluysian atrophy (*ATN1*).

Variants were classified using the guidelines for variant classification of the American College of Medical Genetics and Genomics (ACMG) and the Association of Molecular Pathology [28] and we assigned variants to groups based on disease association [29].

## Results

Through the PAN study, we identified 221 patients with a disease duration of over 4 years, which was characterized by isolated UMN loss in at least 2 regions. After reviewing medical records, we excluded 7 cases in which there was uncertainty about the diagnosis. In 11 patients WES had already been performed in a diagnostic setting and this data was used for analysis in this study. For the remaining 202 patients DNA samples were present, of which only 128 were of sufficient quality and were analysed using WES. Therefore, WES data was available for a total of 139 cases. In 129 of these 139 cases repeat-primed PCR for *C9orf72* was also performed. The baseline characteristics at the time of diagnosis for the patients that were sequenced are presented in Table 1. 14 of 139 cases had UMN dysfunction in only one region at the time of diagnosis, but this progressed to involvement in more than two regions during follow-up visits, therefore meeting the Turner criteria for PLS. In some cases minimal electromyographic (EMG) changes were identified, but these are the “‘low-grade’, non-progressive EMG signs of limited muscle denervation” that are permitted within the Turner criteria [8].

**Table 1:** Baseline characteristics

PLS patients (*n*)	139
Male, *n* (%)	79 (57%)
Mean age of onset yrs (range)	56 (22–83)
Mean age at diagnosis yrs (range)	61 (32–87)
Mean disease duration, months (range)*	
Deceased *n* = 50	161 = 13.4 years (51–504)
Alive *n* = 89	184 = 15.4 years (83–511)
Site of onset, *n* (%)	
Respiratory	0 (0%)
Bulbar	28 (20%)
Spinal	111 (80%)
Arms	8
Legs	103
Cognitive and/or behavioural changes, *n* (%)	
None	102 (73%)
Diagnosis FTD	3 (2%)
Cognitive and/or behavioural impairment [30]	8 (6%)
Pseudobulbar affect	17 (12%)
Missing	9 (7%)
Family history, *n* (%)	
Negative	110 (79%)
Positive for MND	4 (3%)
Suspect for HSP/CMT	4 (3%)
Other	
Parkinson’s disease	7 (5%)
Multiple sclerosis	2 (1%)
Missing	12 (9%)

All characteristics were collected from the moment of diagnoses or within a 3-month time-interval after diagnosis

CMT Charcot–Marie–Tooth, FTD frontotemporal dementia, HSP hereditary spastic paraplegia, MND motor neuron disease, n number, PLS primary lateral sclerosis, UMN upper motor neuron

Pseudobulbar affect includes emotional lability with involuntary laughing/crying/yawning

*For the patients that deceased (*n* = 59), mean disease duration was date of onset—date of death, for patients that are still alive this was date of onset—26-08-2022

In our cohort a total of 4 patients had a positive family history of MND, and 4 patients were classified as suspect for HSP/CMT (pes cavus and tripping easily, pes cavus and hammertoes, walking problems) without a clear family history or genetic variant known in the family. In three of these cases, a pathogenic or likely pathogenic variants was identified. One patient with a family member with ALS had a *C9orf72* repeat expansion (case 1) and 2 cases that had a family history suspect for HSP/CMT had a pathogenic variant in *TBK1*, which is associated with ALS-FTD. In the five other cases with a positive family history no relevant genetic variants were identified.

Genetic analyses (WES and repeat-primed PCR for *C9orf72*) revealed variants in 31 out of 139 cases (22%). There were 7 cases with one/two pathogenic variant(s), 4 cases with likely pathogenic variants and 20 cases with variants of uncertain significance (VUS) [28]. Genetic and clinical characteristics of these cases are presented in Table 2.

**Table 2:** PLS cases with a genetic variant

	Genetic information						Clinical information							
	Gene	Variant (RNA/protein/ splicing change)	Classification (ACMG)	Inheritance	gnomAD allele count	ACMG details	Sex	AoO/ AoD (y)	DD (y)	BS	UU	Sensory symptoms	Non-motor symptoms	Family history
1	C9orf72	> 30 repeats (pathogenic > 30)	Pathogenic	Heterozygous	NA	NA	M	39/45	17.2*	+	+	Numbness of both feet	PBA	Niece of father had ALS
2	C9orf72	> 30 repeats (pathogenic > 30)	Pathogenic	Heterozygous	NA	NA	M	71/77	10.8	+	Unk	–	PBA, difficulty remembering names and people from the past, short term memory loss	–
3	SPAST	whole gene deletion	Pathogenic	Heterozygous	NA	NA	F	31/35	42	–	–	–	Pes cavus and hammer toes	Unk
4	SPG11 SPG11	r.spl c.7000-2del p.(Gln2301*)	Pathogenic Pathogenic	Heterozygous Heterozygous	– –	PVS1, PM2, PM3 PVS1, PM2, PM3	F	73/75	9.1	–	+	–	–	–
5	SPG7	p.(Leu78*)	Pathogenic	Homozygous	112/282546	PVS1, PM3,	F	31/39	37.3*	–	–	–	–	–
6	TBK1	p.(Gly217Arg)	Pathogenic	Heterozygous	–	PS3, PS4, PP3	F	66/67	6.2	–	–	–	PBA	Son walking problems
7	TBK1	p.(Gly217Arg)	Pathogenic	Heterozygous	–	PS3, PS4, PP3	M	75/77	5.2	+	+	–	PBA, pes cavus	Pes cavus and hammer toes
8	FIG4	p.(Ile41Thr)	L Pathogenic (recessive)	Heterozygous	284/282242	PS3, PM3, PP3 (recessive)	M	52/57	9.5	–	+	–	–	–
9	NEFL	p.(Thr88Pro)	L Pathogenic	Heterozygous	–	PS4, PP1, PP3	M	53/56	11.7*	–	–	–	EMG: axonal sensorimotor polyneuropathy	–
10	SPG7 SPG7	p.(Ala510Val) p.(Ile743Thr)	L Pathogenic L Pathogenic	Heterozygous Heterozygous	820/282858 14/282720	PS4, PM3, PP1, PP3 PS4, PM3, PP1, PP3	M	37/40	31.5	–	–	–	PBA	Unk
11	TBK1	r.spl c.87 G > A	L Pathogenic	Heterozygous	–	PVS1, PM2	F	62/65	4.3	+	+	–	PBA, prosopagnosia	–
12	ABCD1	p.(Glu421Lys)	VUS	Hemizygous	1/183147	PM2	M	46/47	9.8	+	–	–	–	–
13	ALDH18A1	p.(Arg732Cys)	VUS	Heterozygous	2/282722	PP3	M	60/61	7.9*	–	–	Numb right foot	–	–
14	ANG	p.(His37Arg)	VUS	Heterozygous	3/251494	PP3	M	49/57	19.1*	+	–	–	–	–
15	ANXA11	p.(Val352Met)	VUS	Heterozygous	6/251416	PP3	M	38/44	25.1*	–	+	–	–	–
16	ATXN2	32 repeats (normal < 32; pathogenic > 34)	VUS	Heterozygous	NA	NA	F	71/74	9.9	–	–	–	PBA	–
17	CHMP2B	p.(Met102Val)	VUS	Heterozygous	1/31388	PP3	M	58/59	19.3	–	–	–	FTD	–
18	DCTN1	p.(Asp748Glu)	VUS	Heterozygous	5/282770	PP3	M	61/64	11.1*	+	+	–	PBA	–
19	ERBB4	p.(Arg484Lys)	VUS	Heterozygous	2/282866	PP3	F	51/52	14.2*	–	–	–	–	–
20	FA2H	p.(Val309Ile)	VUS	Homozygous	60/282546	PP3	M	58/63	8.5	+	–	–	–	–
21	FUS	p.(Gln179His)	VUS	Heterozygous	–	PM2, PP3	M	65/71	24.8*	–	–	–	–	–
22	FUS	p.(Gly474Arg)	VUS	Heterozygous	1/250650	PP3	F	62/67	11.7	–	+	Numbness and lowered vibration sense left leg	–	–
23	NEK1	p.(Gln95*)	VUS	Heterozygous	1/248912	PVS1,	F	69/71	5.8	+	–	–	–	–
24	NEK1	p.(Ser1047Leu)	VUS	Heterozygous	7/280336	PP3	M	77/81	13.8	+	–	–	PBA	–
25	NIPA1	p.(Ala86Gly)	VUS	Hemizygous	–	PM2, PP3	M	39/49	10.3	–	–	–	–	–
26	OPTN	p.(Leu410Pro)	VUS	Heterozygous	3/250750	PP3	M	47/48	9*	+	–	–	CTS bilaterally + ulnar neuropathy left (both improved after CTS treatment)	–
27	PPP2R2B	46 repeats (normal < 33; pathogenic > 50)	VUS	Heterozygous	NA	NA	F	56/61	15.5*	–	Unk	Numbness of distal limbs	PBA	–
28	SETX	p.(His227Leu)	VUS	Heterozygous	–	PM2, PP3	M	83/87	7.8	+	–	–	–	–
29	UBAP1	p.(His430Tyr)	VUS	Heterozygous	3/282828	PP3	M	64/65	12.3	–	–	Tingling of distal limbs, numbness during walking	Unk	–
30	VCP	p.(Ile114Val)	VUS	Heterozygous	20/282856	PP3	M	51/53	13.6*	–	–	–	Pes cavus and hammer toes	–
31	ZFYVE27	r.(spl?) c.912 + G > A	VUS	Heterozygous	–	PM2, PP3	M	52/68	29.8	+	+	–	PBA	–

Evidence of pathogenicity was determined using the ACMG criteria for classifying pathogenic variants

PVS1: very strong evidence of pathogenicity (null variant in a gene where loss of function is a known mechanism of disease); PS3: strong evidence of pathogenicity (well-established in vitro or in vivo functional studies supportive of a damaging effect on the gene or gene product; PS4: strong evidence of pathogenicity (the prevalence of the variant in affected individuals is significantly increased compared to the prevalence in controls); PM2: moderate evidence of pathogenicity (absent from controls or at extremely low frequency if recessive); PM3: moderate evidence of pathogenicity (for recessive disorders, detected in *trans* with a pathogenic variant); PM4: moderate evidence of pathogenicity (protein length changes due to in-frame deletions/insertions in a non-repeat region or stop-loss variants; PP1: supporting evidence of pathogenicity (co-segregation with disease in multiple affected family members in a gene definitively known to cause a disease); PP3: supporting evidence of pathogenicity (multiple lines of computational evidence support a deleterious effect on the gene or gene product)

ACMG American College of Medical Genetics and Genomics, AoD age of diagnosis, AoO age of onset, BS Bulbar symptoms, DD disease duration (was date of onset– date of death, for patients that are still alive* in our database this was date of diagnosis—26-8-2022), EMG electromyography, F female, FTD frontotemporal dementia, L likely, M male, NA not applicable, PBA pseudobulbar affect (emotional inhibition, inappropriate laughing and/or crying and/or yawning), RNA ribonucleic acid, Unk unknown, UU urinary urgency, VUS variant of unknown significance, y years, +  present, − absent

**Fig. 1:**
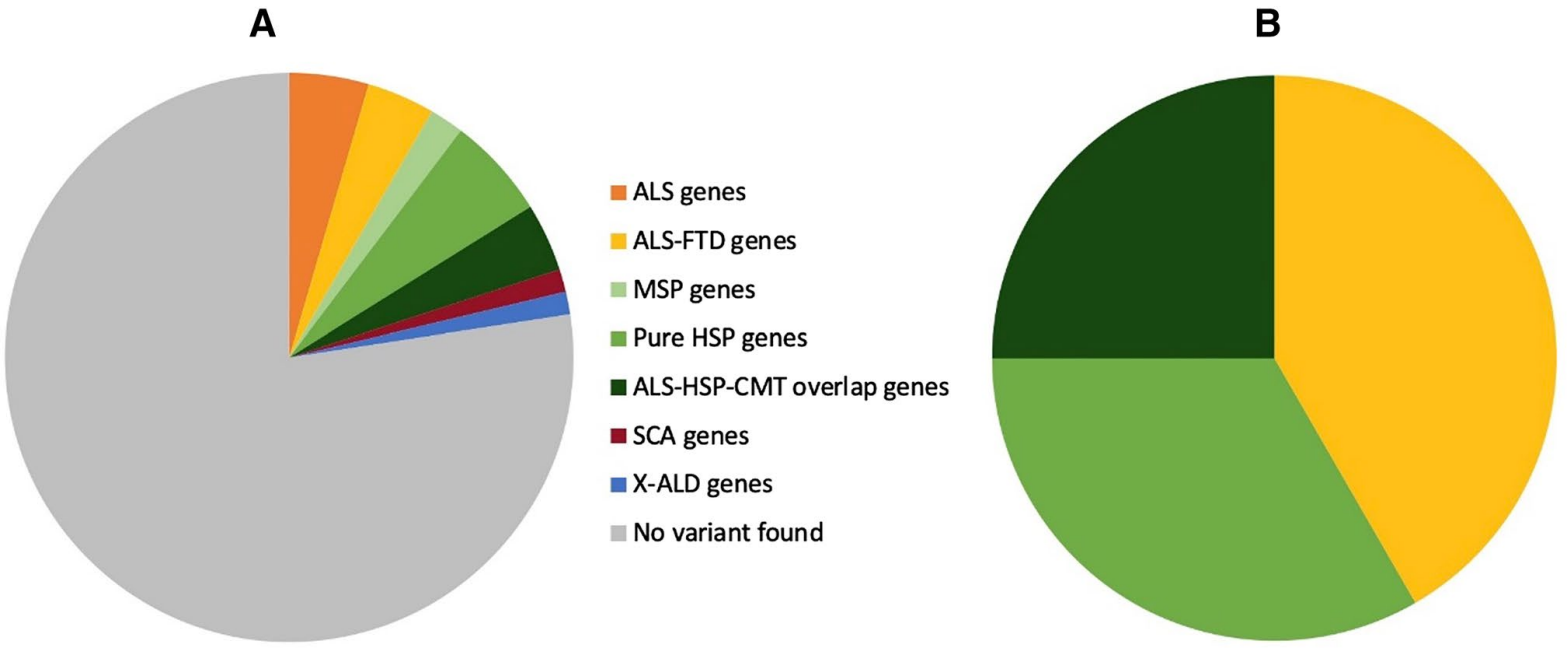
Distribution of genetic variants found. Phenotypes were determined using the gene database of the National Centre for Biotechnology Information on 25-08-2022 [29]. **A** Whole cohort with all variants found; **B** pathogenic and likely pathogenic variants. ALS genes: *FUS, NEK1, ANG, ERBB4*; ALS-FTD genes: *C9orf72, TBK1, CHMP2B*; pure HSP genes: *SPG7, SPAST, ZFVE27, FA2H, UBAP1, ALDH18A1*; MSP genes: *VCP, OPTN, ANXA11*; “ALS-HSP-CMT overlap” genes: *SPG11, DCTN1, FIG4, SETX, NIPA1, NEFL*; SCA genes: *ATXN2, PPP2R2B*; X-ALD genes: *ABCD1.* ALS amyotrophic lateral sclerosis, CMT Charcot–Marie–Tooth, FTD frontotemporal dementia, HSP hereditary spastic paraplegia, MSP multisystem proteinopathy, SCA spinocerebellar ataxia, X-ALD X-linked adrenoleukodystrophy

Figure 1A shows the distribution of genetics variants grouped by disease association, which includes pathogenic, likely pathogenic variants and variants of uncertain significance, whereas Fig. 1B only shows pathogenic and likely pathogenic variants. Case 8 was heterozygous for a variant in *FIG4* that is likely pathogenic in a homozygous state. We therefore consider this to be a VUS.

In total, 10 cases (accounting for 7% of the total cohort) were found to a carry pathogenic or likely pathogenic variant; 5 cases had an ALS-FTD gene variant (50%), 3 cases pure HSP gene variant (33%) and 2 cases an ALS-HSP-CMT gene variant (20%). Characteristics of these groups are presented in Table 3.

**Table 3:** Pathogenic/likely pathogenic variants clustered by disease association

	ALS-FTD group *C9orf72; TBK1*	Pure HSP group *SPAST; SPG7*	ALS-HSP-CMT group *NEFL; SPG11*
Patients (*n*)	5	3	2
Mean age of onset, yrs. (range)	62.6 (39–77)	33 (31–37)	63 (53–73)
Mean disease duration, yrs. (range)	8.7 (4.3–17.2)	36.9 (31.5–42)	10.4 (9.1–11.7)
Site of onset	2/5 bulbar, 3/5 LE	3/3 LE	2/2 LE
Bulbar symptoms	4/5	0/3	0/2
Involvement UE (% asymmetry)	5/5 (80%)	3/3 (0%)	1/2 (50%)
Involvement LE (% asymmetry)	5/5 (60%)	3/3 (0%)	2/2 (50%)
Pseudobulbar affect	5/5	1/3	0/2
Cognitive changes	2/5	0/3	0/2

Mean disease duration is based on the disease duration presented in Table 2

ALS amyotrophic lateral sclerosis, CMT Charcot–Marie–Tooth, FTD frontotemporal dementia, HSP hereditary spastic paraplegia, LE lower extremities, UE upper extremities

In our initial cohort of patients with a disease duration longer than 4 years, we also had 9 patients that presented with isolated UMN signs in one single region (2 bulbar and 7 lower limbs). For two patients, we unfortunately had no clinical follow-up data and for the other 7 patients the symptoms remained within one region. These 9 patients did not meet the Turner criteria for PLS and are therefore not included in the results. They did have genetic analyses as mentioned above (9 WES, 8 *C9orf72*), which resulted in one patient with a likely pathogenic homozygous variant in *SPG7* (male, onset lower extremities at 30 years old, disease duration of 36 years), one patient with a heterozygous VUS in *REEP1* (male, onset lower extremities at 64 years old, disease duration of 15 years) and one with a hemizygous VUS in *UBQLN2* (male, onset bulbar at 65 years old, disease duration of 8 years).

We found two cases to be compound heterozygous for variants in *SPG11* (case 4) and in *SPG7* (case 10).

Two patients carried identical *TBK1* variants (case 6 and 7), but based on family history, family name and geographical origin we were not able to detect any family tie.

In 1 case (case 12) a variant in *ABCD1* was found. According to the ACMG criteria this is classified as a VUS and the phenotype (history and MRI findings) did not match X-ALD. Unfortunately, C26:0-lysophosphatidylcholine (marker for very long-chain fatty acid accumulation in phospholipid fraction) analyses was not possible because the patients had died and therefore we were unable to definitely reject the diagnosis X-ALD on biochemical grounds [31].

In three of the eight patients that reported a positive or suspect family history for either MND or HSP/CMT phenotypes, a pathogenic or likely pathogenic variant was identified (cases 1, 6 and 7). These were a *C9orf72* repeat expansions in a case where a distant relative (niece of father) had been diagnosed with ALS and two cases of a *TBK1* variant (son with walking problems and foot deformities in the family). In the remaining five cases with a positive or suspect family history no relevant variants were identified.

## Discussion

Current consensus criteria for PLS state that there is limited or no place for genetic testing in the work-up for PLS [8]. However, a very limited number of studies has been conducted on the genetics of PLS and as a result the data supporting this statement is sparse.

In an attempt to clarify what the contribution of genetics is to PLS susceptibility, Silani and colleagues reviewed the literature [32]. They note that in previous diagnostic criteria (Pringle) a negative family history was required [6], which was mainly meant to differentiate between PLS and HSP. However, in later criteria this requirement was dropped, as rare pedigrees with multiple PLS cases have been reported. This review identified reports of *C9orf72* repeat expansions in 3 PLS patients and single cases with mutations in *FIG4*, *UBQLN2*, *OPTN* and *DCTN1*. They conclude that the finding of ALS mutations in PLS cases is rare, but also acknowledge that study sizes were limited [32].

The largest of these studies was performed by van Rheenen, et al. (*n* = 110), but only analyzed *C9orf72* repeat expansions [25]. The only other relatively large study was performed by Mitsumoto, et al. (*C9orf72* and exome sequencing), which included 34 PLS patients with disease duration of > 5 years. This study found 18% of patients to carry a variant in either ALS (*C9orf72)*, Parkinson disease (*PARK2, LRRK2*) or HSP (*SPG7*) genes [33]. All other studies had sample sizes smaller than 10 or were case reports on single cases or pedigrees [32]. Therefore, firm conclusions regarding the frequency of pathogenic or likely pathogenic variants in PLS cannot be reached.

In our cohort, which is the largest to date, we found genetic variants in ALS or movement disorder genes in 31 out of 139 cases (22%). It must, however, be noted that a considerable portion of these (*n* = 21) are variants of unknown significance. It is likely that some of these variants are not clinically relevant, such as the *ABCD1* variant we identified.

To provide better interpretation of variants of unknown significance, it remains crucial that they are reported in clinical and scientific reports. Only by doing so, will evidence accumulate over time and therefore allow us to accurately interpret the clinical relevance of these variants. Likely pathogenic or pathogenic variants were found in 7% of our cohort. These findings raise the complicated question of how to diagnostically classify the cases carrying (pleiotropic) ALS and HSP/CMT variants. For example, in case 9 in which a *NEFL* variant was identified, which is associated with CMT [34], and HSP [35]. This specific patient had bulbar and upper extremity involvement and met the criteria for definite PLS, making the clinical phenotype more compatible with PLS than HSP according to the treating physicians. According to the current consensus criteria genetic testing would not be warranted in this patient, while we believe that based on these genetic results the phenotype should be considered HSP-plus rather than PLS.

The revised El Escorial criteria for ALS state that patients with progressive upper and/or lower motor neuron signs and a family history of a defined pathogenic ALS mutation meet the criteria for “clinically definite familial ALS – Laboratory-supported” [36]. This means only progressive upper motor neuron signs in combination with a mutation in an ALS gene suffices. Therefore, it would seem reasonable to diagnose patients with PLS phenotypes carrying ALS variants as atypical or slow forms of ALS.

The Turner criteria state that testing for HSP genes should only be considered in patients with symmetrical involvement of the lower limbs [8]. Although it is uncommon, HSP patients with one-sided symptoms have been described [14, 15], and this is also seen in 2 of our cases with a (likely) pathogenic variant (“ALS-HSP-CMT overlap group”). It is even relatively common for patients with pure HSP to experience some degree of asymmetry of severity of symptoms in their legs [37, 38]. Apart from asymmetry, arm involvement (so UMN involvement in two regions) has also been documented in HSP patients [13, 39], as well as dysarthria which is reported frequently [39]. A study by Brugman et al. studied whether it was possible to differentiate sporadic HSP from PLS based on clinical presentation and concluded that this is unreliable in most cases [40]. Similarly, it seems reasonable to diagnose patients with PLS phenotypes carrying HSP variants as atypical forms of HSP.

Not all patients with a positive family history for MND or suspect for HSP/CMT in our cohort had a genetic variant identified. Despite the negative genetic findings in these cases, the family history is suggestive of a genetic cause of the disease. Indeed, it is not uncommon that in cases of HSP (also with a positive family history) the disease-causing gene is not found. Mereaux et al. performed genetic testing in a large cohort of HSP cases (1550) and pathogenic/likely pathogenic variants were found in 23.9–54.2% of the familial cases (variating between inheritance) and 26.6% of the isolated cases [16]. 5–15% of the ALS cases is familial and in 60–80% a disease-causing variant can be found (mostly *C9orf72*, *SOD1*, *FUS* and *TARDBP*) [2]. Therefore, our current knowledge of HSP and ALS genetics is incomplete. However, progress in genetic research is occurring at an ever-increasing pace and it seems highly likely that additional novel HSP and ALS genes will be discovered over the next few years. It also seems highly likely that variants in these genes will also be identified in PLS cases.

The diagnostic yield of genetic testing for HSP and ALS genes in PLS is 7% based on our findings.

We do believe that based on the results of Mitsumoto et al. [33] and our cohort, genetic analyses could have a role in the diagnostic work-up of PLS and should not be limited to patients with only symmetrical involvement of the legs.

Incorporating genetic testing in the work-up of patients with primary pyramidal disorders could lead to clarity regarding the underlying diagnosis for individual patients. Furthermore, it may also provide patients with additional information with regards to their prognosis. There are substantial differences between ALS, PLS and HSP in the rate of disease progression, degree of disability the disease will eventually cause as well as in life-expectancy. Although, this will become apparent over time, in the early stages of the disease this is frequently not clear, causing uncertainty and distress to patients. A desire for pregnancy (among relatives) may also be a reason to have clarity on whether there is a hereditary cause of the disease.

As the prices for DNA sequencing have dramatically dropped over the last couple of years, it is to be expected that genetic testing will become more widely available in the nearby future. DNA testing may, however, also yield results that are difficult to interpret, such as variants of unknown significance, and thereby provide the opposite of clarity. Genetic testing should therefore only be requested by skilled physicians with an up-to-date knowledge of the genetics of motor neuron diseases, who are capable of interpreting and adequately communicating results to patients. An infrastructure to provide genetic counselling to at risk family members should also be in place, to ensure that complicated topics such as mode of inheritance, disease penetrance and prenatal diagnostic testing are discussed appropriately. The decision to perform genetic testing in clinical practice is complex and should, in our opinion always be reached through shared decision making.

This paper offers an in-depth genetic characterization of the largest PLS cohort to date. Our findings show that patients with clinical phenotypes compatible with PLS may carry (likely) pathogenic variants in ALS and/or HSP genes. This raises the question whether PLS is a distinct disease entity or represents endophenotypes within the spectrum of different diseases such as ALS and HSP? The fact that there is no gold standard for PLS complicates this even further.

A limited number of histopathological reports on PLS patients are available, of which some predate the identification of TDP-43. Findings have been heterogeneous and perhaps these publications contain a bias towards unusual cases [41]. Of note, however are recent post-mortem studies by Mackenzie et al. that demonstrated TDP-43 pathology and degeneration of upper motor neurons, with preservation of lower motor neurons and less TDP-43 pathology in seven PLS cases [42]. The addition of a histopathological gold standard to the formal criteria for PLS would constitute a major step forward and should be focused on future efforts.

Similarly prospective data, including genetic results, on larger, international cohorts of PLS patients would also provide additional clarity. Potentially, genome-wide association studies or whole-genome sequencing studies of international PLS cohorts could lead to the identification of novel disease genes or phenotypic modifiers, that are associated with PLS. Understanding the underlying pathophysiology of PLS would be major step forward and could potentially guide therapy development, such as antisense based approaches.

## Supplementary Information

Below is the link to the electronic supplementary material.

Supplementary file1 (PDF 386 KB)

Supplementary file2 (PDF 111 KB)
