# Supplementary material for: Genetic characterization of primary lateral sclerosis
Source: J Neurol. 2023 May 3;270(8):3970–80. doi: 10.1007/s00415-023-11746-7 (PMC10345048; doi:10.1007/s00415-023-11746-7)
Supplement: Supplementary file 1 — Supplementary file1 (PDF 386 KB) [file 415_2023_11746_MOESM1_ESM.pdf]

MOVEMENT DISORDERS GENE PANEL DG 2.18 (338 genes)

Releasedate: 20-04-2020

Gene	Agilent V5 covered >10x	Agilent V5 covered > 20x	TWIST covered >10x	TWIST covered >20x	Associated Phenotype description and OMIM disease ID
AARS2	100%	99,40%	100%	100%	Combined oxidative phosphorylation deficiency 8, 614096<br>Leukoencephalopathy, progressive, with ovarian failure, 615889
ABCB7	99,50%	98,20%	99,80%	99,30%	Anemia, sideroblastic, with ataxia, 301310
ABCD1	75,80%	71,60%	100%	100%	Adrenomyeloneuropathy, adult, 300100<br>Adrenoleukodystrophy, 300100
ABHD12	98,70%	92,30%	100%	99,30%	Polyneuropathy, hearing loss, ataxia, retinitis pigmentosa, and cataract, 612674
ACTB	99,70%	96,10%	100%	100%	?Dystonia, juvenile-onset, 607371<br>Baraitser-Winter syndrome 1, 243310
ADAR	100%	99,80%	100%	100%	Aicardi-Goutieres syndrome 6, 615010<br>Dyschromatosis symmetrica hereditaria, 127400
ADCY5	95,10%	91,20%	99,20%	98,00%	Dyskinesia, familial, with facial myokymia, 606703
ADGRG1	100%	100%	100%	100%	Polymicrogyria, bilateral perisylvian, 615752<br>Polymicrogyria, bilateral frontoparietal, 606854
ADPRHL2	100%	99,80%	100%	100%	Neurodegeneration, childhood-onset, stress-induced, with variable ataxia and seizures, 618170
AFG3L2	95,00%	91,10%	100%	99,90%	Spastic ataxia 5, autosomal recessive, 614487<br>Spinocerebellar ataxia 28, 610246
AGTPBP1	96,00%	94,10%	100%	100%	Neurodegeneration, childhood-onset, with cerebellar atrophy, 618276
AIMP1	99,20%	94,50%	100%	99,90%	Leukodystrophy, hypomyelinating, 3, 260600
ALDH18A1	100%	99,90%	100%	100%	Cutis laxa, autosomal recessive, type IIIA, 219150<br>Cutis laxa, autosomal dominant 3, 616603<br>Spastic paraplegia 9B, autosomal recessive, 616586<br>Spastic paraplegia 9A, autosomal dominant, 601162
ALDH3A2	95,30%	94,60%	100%	100%	Sjogren-Larsson syndrome, 270200
ALS2	100%	99,90%	100%	100%	Primary lateral sclerosis, juvenile, 606353<br>Amyotrophic lateral sclerosis 2, juvenile, 205100<br>Spastic paralysis, infantile onset ascending, 607225
AMPD2	99,80%	98,90%	100%	100%	?Spastic paraplegia 63, 615686<br>Pontocerebellar hypoplasia, type 9, 615809
ANO10	99,80%	97,90%	100%	100%	Spinocerebellar ataxia, autosomal recessive 10, 613728
ANO3	99,50%	97,80%	100%	100%	Dystonia 24, 615034

AP4B1	99,90%	98,70%	100%	100%	Spastic paraplegia 47, autosomal recessive, 614066
AP4E1	99,80%	98,70%	100%	100%	Stuttering, familial persistent, 1, 184450<br>Spastic paraplegia 51, autosomal recessive, 613744
AP4M1	99,90%	98,90%	100%	100%	Spastic paraplegia 50, autosomal recessive, 612936
AP4S1	78,90%	71,30%	87,90%	87,90%	Spastic paraplegia 52, autosomal recessive, 614067
APTX	94,90%	92,50%	100%	100%	Ataxia, early-onset, with oculomotor apraxia and hypoalbuminemia, 208920
ARG1	100%	100%	100%	100%	Argininemia, 207800
ARSA	100%	99,80%	100%	100%	Metachromatic leukodystrophy, 250100
ARX	81,00%	64,00%	91,50%	85,70%	Proud syndrome, 300004<br>Partington syndrome, 309510<br>Lissencephaly, X-linked 2, 300215<br>Epileptic encephalopathy, early infantile, 1, 308350<br>Mental retardation, X-linked 29 and others, 300419<br>Hydranencephaly with abnormal genitalia, 300215
ASPA	99,90%	98,30%	100%	100%	Canavan disease, 271900
ATCAY	100%	99,80%	100%	100%	Ataxia, cerebellar, Cayman type, 601238
ATL1	100%	99,70%	100%	100%	Spastic paraplegia 3A, autosomal dominant, 182600<br>Neuropathy, hereditary sensory, type ID, 613708
ATM	99,80%	98,10%	100%	100%	Ataxia-telangiectasia, 208900<br>Lymphoma, mantle cell, somatic, 0<br>Lymphoma, B-cell non-Hodgkin, somatic, 0<br>T-cell prolymphocytic leukemia, somatic, 0
ATP13A2	100%	99,50%	100%	100%	Kufor-Rakeb syndrome, 606693<br>Spastic paraplegia 78, autosomal recessive, 617225
ATP1A2	100%	100%	100%	100%	Migraine, familial hemiplegic, 2, 602481<br>Migraine, familial basilar, 602481<br>Alternating hemiplegia of childhood 1, 104290
ATP1A3	100%	99,90%	100%	100%	CAPOS syndrome, 601338<br>Alternating hemiplegia of childhood 2, 614820<br>Dystonia-12, 128235
ATP2B3	99,50%	97,50%	100%	100%	?Spinocerebellar ataxia, X-linked 1, 302500
ATP7B	99,90%	99,20%	100%	100%	Wilson disease, 277900
B4GALNT1	99,30%	95,00%	100%	100%	Spastic paraplegia 26, autosomal recessive, 609195
BCAP31	92,60%	83,20%	100%	99,90%	Deafness, dystonia, and cerebral hypomyelination, 300475
BCKDHA	99,90%	99,20%	100%	100%	Maple syrup urine disease, type Ia, 248600
BCKDHB	99,50%	94,40%	100%	100%	Maple syrup urine disease, type Ib, 248600

BCL11B	99,10%	95,60%	98,80%	97,30%	Immunodeficiency 49, 617237<br>Intellectual developmental disorder with dysmorphic facies, speech delay, and T-cell abnormalities, 618092
BSCL2	100%	100%	100%	100%	Lipodystrophy, congenital generalized, type 2, 269700<br>Silver spastic paraplegia syndrome, 270685<br>Neuropathy, distal hereditary motor, type VA, 600794<br>Encephalopathy, progressive, with or without lipodystrophy, 615924
BTD	100%	99,90%	100%	100%	Biotinidase deficiency, 253260
C12orf65	99,80%	98,50%	100%	100%	Spastic paraplegia 55, autosomal recessive, 615035<br>Combined oxidative phosphorylation deficiency 7, 613559
C19orf12	100%	99,80%	100%	100%	Neurodegeneration with brain iron accumulation 4, 614298<br>?Spastic paraplegia 43, autosomal recessive, 615043
CA8	99,60%	97,30%	100%	100%	Cerebellar ataxia and mental retardation with or without quadrupedal locomotion 3, 613227
CACNA1A	95,10%	91,90%	100%	100%	Spinocerebellar ataxia 6, 183086<br>Epileptic encephalopathy, early infantile, 42, 617106<br>Migraine, familial hemiplegic, 1, with progressive cerebellar ataxia, 141500<br>Episodic ataxia, type 2, 108500<br>Migraine, familial hemiplegic, 1, 141500
CACNA1E	100%	99,90%	100%	100%	Epileptic encephalopathy, early infantile, 69, 618285
CACNA1G	100%	99,60%	100%	100%	Spinocerebellar ataxia 42, 616795<br>Spinocerebellar ataxia 42, early-onset, severe, with neurodevelopmental deficits, 618087
CACNB4	95,50%	94,30%	100%	100%	Episodic ataxia, type 5, 613855
CAMTA1	100%	99,50%	100%	100%	Cerebellar ataxia, nonprogressive, with mental retardation, 614756
CAPN1	100%	100%	100%	100%	Spastic paraplegia 76, autosomal recessive, 616907
CCT5	100%	99,70%	100%	100%	Neuropathy, hereditary sensory, with spastic paraplegia, 256840
CHMP1A	100%	99,80%	100%	100%	Pontocerebellar hypoplasia, type 8, 614961
CLCN2	100%	99,50%	100%	100%	Leukoencephalopathy with ataxia, 615651<br>Hyperaldosteronism, familial, type II, 605635
CLCN4	99,90%	98,90%	100%	100%	Raynaud-Claes syndrome, 300114
CLPB	100%	99,90%	100%	100%	3-methylglutaconic aciduria, type VII, with cataracts, neurologic involvement and neutropenia, 616271
COASY	100%	100%	100%	100%	Pontocerebellar hypoplasia, type 12, 618266<br>Neurodegeneration with brain iron accumulation 6, 615643
COL4A1	98,70%	97,40%	100%	100%	?Retinal arteries, tortuosity of, 180000<br>Brain small vessel disease with or without ocular anomalies, 175780<br>Angiopathy, hereditary, with nephropathy, aneurysms, and muscle cramps, 611773<br>Microangiopathy and leukoencephalopathy, pontine, autosomal dominant, 618564
COL6A1	100%	99,40%	100%	100%	Ullrich congenital muscular dystrophy 1, 254090<br>Bethlem myopathy 1, 158810

COL6A2	100%	99,80%	100%	100%	Bethlem myopathy 1, 158810<br>?Myosclerosis, congenital, 255600<br>Ullrich congenital muscular dystrophy 1, 254090
COL6A3	100%	99,80%	100%	100%	Bethlem myopathy 1, 158810<br>Dystonia 27, 616411<br>Ullrich congenital muscular dystrophy 1, 254090
COQ2	98,00%	95,30%	97,20%	97,20%	Coenzyme Q10 deficiency, primary, 1, 607426
COQ4	90,90%	89,30%	100%	100%	Coenzyme Q10 deficiency, primary, 7, 616276
COQ8A	100%	99,50%	100%	100%	Coenzyme Q10 deficiency, primary, 4, 612016
COQ9	100%	97,90%	100%	100%	Coenzyme Q10 deficiency, primary, 5, 614654
COX20	97,80%	88,30%	100%	100%	Mitochondrial complex IV deficiency, 220110
CP	94,80%	88,90%	100%	100%	Hemosiderosis, systemic, due to aceruloplasminemia, 604290<br>Cerebellar ataxia, 604290
CSF1R	99,90%	99,30%	100%	100%	Brain abnormalities, neurodegeneration, and dysosteosclerosis, 618476<br>Leukoencephalopathy, diffuse hereditary, with spheroids, 221820
CSTB	99,60%	89,80%	100%	100%	Epilepsy, progressive myoclonic 1A (Unverricht and Lundborg), 254800
CYP27A1	98,90%	96,70%	100%	100%	Cerebrotendinous xanthomatosis, 213700
CYP2U1	94,80%	91,50%	100%	99,90%	Spastic paraplegia 56, autosomal recessive, 615030
CYP7B1	98,00%	92,80%	100%	100%	Spastic paraplegia 5A, autosomal recessive, 270800<br>Bile acid synthesis defect, congenital, 3, 613812
DARS2	100%	99,30%	100%	100%	Leukoencephalopathy with brain stem and spinal cord involvement and lactate elevation, 611105
DBT	99,80%	98,00%	100%	100%	Maple syrup urine disease, type II, 248600
DCAF17	98,90%	93,30%	100%	100%	Woodhouse-Sakati syndrome, 241080
DCC	100%	100%	100%	100%	Esophageal carcinoma, somatic, 133239<br>Gaze palsy, familial horizontal, with progressive scoliosis, 2, 617542<br>Mirror movements 1 and/or agenesis of the corpus callosum, 157600<br>Colorectal cancer, somatic, 114500
DCTN1	100%	98,80%	100%	100%	Perry syndrome, 168605<br>Neuronopathy, distal hereditary motor, type VIIB, 607641
DDC	99,70%	96,40%	100%	100%	Aromatic L-amino acid decarboxylase deficiency, 608643
DDHD1	97,90%	95,80%	100%	100%	Spastic paraplegia 28, autosomal recessive, 609340
DDHD2	100%	99,60%	100%	100%	Spastic paraplegia 54, autosomal recessive, 615033
DHDDS	99,00%	95,00%	95,20%	95,20%	Retinitis pigmentosa 59, 613861<br>?Congenital disorder of glycosylation, type 1bb, 613861<br>Developmental delay and seizures with or without movement abnormalities, 617836
DLAT	100%	99,70%	100%	100%	Pyruvate dehydrogenase E2 deficiency, 245348
DLD	100%	99,70%	100%	100%	Dihydrolipoamide dehydrogenase deficiency, 246900
DNAJC12	87,40%	87,40%	100%	100%	Hyperphenylalaninemia, mild, non-BH4-deficient, 617384

DNAJC3	100%	99,70%	100%	100%	?Ataxia, combined cerebellar and peripheral, with hearing loss and diabetes mellitus, 616192
DNAL4	100%	98,90%	100%	100%	?Mirror movements 3, 616059
DNM1L	99,90%	98,50%	100%	100%	Encephalopathy, lethal, due to defective mitochondrial peroxisomal fission 1, 614388<br>Optic atrophy 5, 610708
DNMT1	99,20%	99,00%	99,70%	99,20%	Neuropathy, hereditary sensory, type IE, 614116<br>Cerebellar ataxia, deafness, and narcolepsy, autosomal dominant, 604121
DPYS	100%	99,90%	100%	100%	Dihydropyrimidinuria, 222748
ECHS1	99,90%	99,00%	100%	100%	Mitochondrial short-chain enoyl-CoA hydratase 1 deficiency, 616277
EIF2B1	100%	100%	100%	100%	Leukoencephalopathy with vanishing white matter, 603896
EIF2B2	99,90%	99,50%	100%	100%	Ovarioleukodystrophy, 603896<br>Leukoencephalopathy with vanishing white matter, 603896
EIF2B3	100%	100%	100%	100%	Leukoencephalopathy with vanishing white matter, 603896
EIF2B4	100%	99,90%	100%	100%	Leukoencephalopathy with vanishing white matter, 603896<br>Ovarioleukodystrophy, 603896
EIF2B5	100%	99,00%	100%	100%	Leukoencephalopathy with vanishing white matter, 603896<br>Ovarioleukodystrophy, 603896
ELOVL4	100%	99,50%	100%	100%	Spinocerebellar ataxia 34, 133190<br>Stargardt disease 3, 600110<br>Ichthyosis, spastic quadriplegia, and mental retardation, 614457
ELOVL5	100%	99,80%	100%	100%	Spinocerebellar ataxia 38, 615957
ERLIN2	100%	99,90%	100%	100%	Spastic paraplegia 18, autosomal recessive, 611225
ETHE1	99,90%	97,40%	100%	100%	Ethylmalonic encephalopathy, 602473
EXOSC3	99,50%	94,90%	100%	100%	Pontocerebellar hypoplasia, type 1B, 614678
EXOSC5	100%	100%	100%	100%	No OMIM disease ID
FA2H	92,00%	83,10%	100%	100%	Spastic paraplegia 35, autosomal recessive, 612319
FAM126A	100%	99,40%	100%	100%	Leukodystrophy, hypomyelinating, 5, 610532
FAR1	97,60%	92,80%	100%	100%	Peroxisomal fatty acyl-CoA reductase 1 disorder, 616154
FARS2	100%	100%	100%	100%	Spastic paraplegia 77, autosomal recessive, 617046<br>Combined oxidative phosphorylation deficiency 14, 614946
FBXO7	99,80%	97,90%	100%	100%	Parkinson disease 15, autosomal recessive, 260300
FGF14	100%	100%	100%	100%	Spinocerebellar ataxia 27, 609307
FLVCR1	100%	98,90%	100%	100%	Ataxia, posterior column, with retinitis pigmentosa, 609033
FOLR1	100%	100%	100%	100%	Neurodegeneration due to cerebral folate transport deficiency, 613068
FRMD7	99,90%	99,10%	100%	99,60%	Nystagmus 1, congenital, X-linked, 310700<br>Nystagmus, infantile periodic alternating, X-linked, 310700

FTL	98,50%	89,40%	100%	100%	Hyperferritinemia-cataract syndrome, 600886<br>Neurodegeneration with brain iron accumulation 3, 606159<br>L-ferritin deficiency, dominant and recessive, 615604
GALC	99,80%	98,30%	100%	100%	Krabbe disease, 245200
GAN	100%	99,60%	100%	100%	Giant axonal neuropathy-1, 256850
GBA	100%	100%	100%	100%	Gaucher disease, type III, 231000<br>Gaucher disease, type IIIC, 231005<br>Gaucher disease, type I, 230800<br>Gaucher disease, perinatal lethal, 608013<br>Gaucher disease, type II, 230900
GBA2	100%	99,70%	100%	100%	Spastic paraplegia 46, autosomal recessive, 614409
GCDH	100%	99,20%	100%	100%	Glutaricaciduria, type I, 231670
GCH1	99,90%	95,50%	100%	100%	Hyperphenylalaninemia, BH4-deficient, B, 233910<br>Dystonia, DOPA-responsive, with or without hyperphenylalaninemia, 128230
GDAP2	100%	99,20%	100%	100%	Spinocerebellar ataxia, autosomal recessive 27, 618369
GFAP	91,80%	89,70%	100%	100%	Alexander disease, 203450
GJC2	78,20%	58,70%	96,90%	91,40%	Spastic paraplegia 44, autosomal recessive, 613206<br>Lymphatic malformation 3, 613480<br>Leukodystrophy, hypomyelinating, 2, 608804
GLB1	99,90%	97,40%	100%	100%	GM1-gangliosidosis, type III, 230650<br>GM1-gangliosidosis, type I, 230500<br>Mucopolysaccharidosis type IVB (Morquio), 253010<br>GM1-gangliosidosis, type II, 230600
GNAL	96,80%	93,40%	100%	100%	Dystonia 25, 615073
GOSR2	95,90%	94,60%	100%	100%	Epilepsy, progressive myoclonic 6, 614018
GPR143	85,80%	76,40%	99,80%	97,90%	Ocular albinism, type I, Nettleship-Falls type, 300500<br>Nystagmus 6, congenital, X-linked, 300814
GRID2	100%	99,80%	100%	100%	Spinocerebellar ataxia, autosomal recessive 18, 616204
GRIN1	100%	100%	100%	100%	Neurodevelopmental disorder with or without hyperkinetic movements and seizures, autosomal recessive, 617820<br>Neurodevelopmental disorder with or without hyperkinetic movements and seizures, autosomal dominant, 614254
GRIN2B	99,80%	99,20%	100%	100%	Epileptic encephalopathy, early infantile, 27, 616139<br>Mental retardation, autosomal dominant 6, 613970
GRM1	100%	99,70%	100%	100%	Spinocerebellar ataxia 44, 617691<br>Spinocerebellar ataxia, autosomal recessive 13, 614831
HACE1	100%	99,30%	100%	100%	Spastic paraplegia and psychomotor retardation with or without seizures, 616756
HEXB	99,60%	96,90%	100%	99,90%	Sandhoff disease, infantile, juvenile, and adult forms, 268800

HK1	100%	100%	100%	100%	Hemolytic anemia due to hexokinase deficiency, 235700<br>Neuropathy, hereditary motor and sensory, Russe type, 605285<br>Neurodevelopmental disorder with visual defects and brain anomalies, 618547<br>Retinitis pigmentosa 79, 617460
HPRT1	99,30%	91,80%	100%	99,30%	HPRT-related gout, 300323<br>Lesch-Nyhan syndrome, 300322
HSD17B4	96,00%	93,70%	96,60%	96,60%	D-bifunctional protein deficiency, 261515<br>Perrault syndrome 1, 233400
HSPD1	98,80%	93,70%	100%	100%	Spastic paraplegia 13, autosomal dominant, 605280<br>Leukodystrophy, hypomyelinating, 4, 612233
IBA57	93,70%	90,10%	100%	100%	?Spastic paraplegia 74, autosomal recessive, 616451<br>Multiple mitochondrial dysfunctions syndrome 3, 615330
MR E11	98,90%	93,30%	100%	100%	Ataxia-telangiectasia-like disorder 1, 604391
ISCA2	100%	98,80%	100%	100%	Multiple mitochondrial dysfunctions syndrome 4, 616370
ITPR1	100%	99,90%	100%	100%	Spinocerebellar ataxia 15, 606658<br>Gillespie syndrome, 206700<br>Spinocerebellar ataxia 29, congenital nonprogressive, 117360
JAM3	100%	99,90%	100%	100%	Hemorrhagic destruction of the brain, subependymal calcification, and cataracts, 613730
KATNB1	100%	99,90%	100%	100%	Lissencephaly 6, with microcephaly, 616212
KCNA1	100%	99,90%	100%	100%	Episodic ataxia/myokymia syndrome, 160120
KCNA2	100%	99,60%	100%	100%	Epileptic encephalopathy, early infantile, 32, 616366
KCNC1	100%	100%	100%	100%	Epilepsy, progressive myoclonic 7, 616187
KCNC3	81,10%	69,40%	94,70%	89,00%	Spinocerebellar ataxia 13, 605259
KCND3	100%	99,40%	100%	100%	Brugada syndrome 9, 616399<br>Spinocerebellar ataxia 19, 607346
KCNJ10	89,30%	89,00%	100%	100%	Enlarged vestibular aqueduct, digenic, 600791<br>SESAME syndrome, 612780
KCNJ6	100%	100%	100%	100%	Keppen-Lubinsky syndrome, 614098
KCNMA1	94,40%	93,60%	100%	100%	Liang-Wang syndrome, 618729<br>Cerebellar atrophy, developmental delay, and seizures, 617643<br>Paroxysmal nonkinesigenic dyskinesia, 3, with or without generalized epilepsy, 609446
KCTD7	95,00%	95,00%	100%	100%	Epilepsy, progressive myoclonic 3, with or without intracellular inclusions, 611726
KIAA1161	100%	100%	100%	100%	Basal ganglia calcification, idiopathic, 7, autosomal recessive, 618317
KIDINS220	100%	100%	100%	100%	Spastic paraplegia, intellectual disability, nystagmus, and obesity, 617296
KIF1A	99,40%	97,10%	100%	100%	NESCAV syndrome, 614255<br>Spastic paraplegia 30, autosomal dominant, 610357

					Neuropathy, hereditary sensory, type IIC, 614213<br>Spastic paraplegia 30, autosomal recessive, 610357
KIF1C	100%	100%	100%	100%	Spastic ataxia 2, autosomal recessive, 611302
KIF5A	100%	99,90%	100%	100%	Myoclonus, intractable, neonatal, 617235<br>Spastic paraplegia 10, autosomal dominant, 604187
KMT2B	95,80%	94,00%	98,70%	97,90%	Dystonia 28, childhood-onset, 617284
L1CAM	99,90%	99,10%	100%	100%	MASA syndrome, 303350<br>Hydrocephalus with Hirschsprung disease, 307000<br>Hydrocephalus with congenital idiopathic intestinal pseudoobstruction, 307000<br>Corpus callosum, partial agenesis of, 304100<br>CRASH syndrome, 303350<br>Hydrocephalus due to aqueductal stenosis, 307000
LAMA1	100%	99,70%	100%	100%	Poretti-Boltshauser syndrome, 615960
LAMB1	100%	99,90%	100%	100%	Lissencephaly 5, 615191
LMNB1	99,90%	98,90%	100%	100%	Leukodystrophy, adult-onset, autosomal dominant, 169500
MAPK8IP3	100%	99,60%	100%	100%	Neurodevelopmental disorder with or without variable brain abnormalities, 618443
MARS2	100%	100%	100%	100%	Spastic ataxia 3, autosomal recessive, 611390<br>?Combined oxidative phosphorylation deficiency 25, 616430
MECP2	100%	98,70%	100%	99,90%	Mental retardation, X-linked syndromic, Lubs type, 300260<br>Encephalopathy, neonatal severe, 300673<br>Mental retardation, X-linked, syndromic 13, 300055<br>Rett syndrome, atypical, 312750<br>Rett syndrome, 312750<br>Rett syndrome, preserved speech variant, 312750
MECR	100%	98,90%	100%	100%	Dystonia, childhood-onset, with optic atrophy and basal ganglia abnormalities, 617282
MFF	94,30%	89,90%	100%	100%	Encephalopathy due to defective mitochondrial and peroxisomal fission 2, 617086
MICU1	98,90%	95,20%	100%	100%	Myopathy with extrapyramidal signs, 615673
MLC1	100%	99,00%	100%	100%	Megalencephalic leukoencephalopathy with subcortical cysts, 604004
MMADHC	94,40%	83,50%	89,70%	89,70%	Homocystinuria, cblD type, variant 1, 277410<br>Methylmalonic aciduria and homocystinuria, cblD type, 277410<br>Methylmalonic aciduria, cblD type, variant 2, 277410
MTHFR	97,30%	96,00%	100%	100%	Homocystinuria due to MTHFR deficiency, 236250
MTPAP	99,50%	96,10%	100%	100%	?Spastic ataxia 4, autosomal recessive, 613672
MTTP	100%	99,60%	100%	100%	Abetalipoproteinemia, 200100
NANS	100%	99,90%	100%	100%	Spondyloepimetaphyseal dysplasia, Camera-Genevieve type, 610442
NEFL	99,90%	98,20%	100%	100%	Charcot-Marie-Tooth disease, type 1F, 607734<br>Charcot-Marie-Tooth disease, dominant intermediate G, 617882<br>Charcot-Marie-Tooth disease, type 2E, 607684

NEU1	99,70%	97,70%	100%	100%	Sialidosis, type II, 256550<br>Sialidosis, type I, 256550
NEXMIF	100%	99,50%	100%	100%	Mental retardation, X-linked 98, 300912
NF2	100%	99,90%	100%	100%	Meningioma, NF2-related, somatic, 607174<br>Schwannomatosis, somatic, 162091<br>Neurofibromatosis, type 2, 101000
NIPA1	100%	100%	99,80%	98,50%	Spastic paraplegia 6, autosomal dominant, 600363
NKX2-1	98,60%	85,60%	100%	100%	Chorea, hereditary benign, 118700<br>Choreoathetosis, hypothyroidism, and neonatal respiratory distress, 610978
NKX6-2	89,00%	81,80%	100%	100%	Spastic ataxia 8, autosomal recessive, with hypomyelinating leukodystrophy, 617560
NOL3	93,70%	84,20%	100%	100%	?Myoclonus, familial, 1, 614937
NPC1	99,60%	98,70%	100%	100%	Niemann-Pick disease, type D, 257220<br>Niemann-Pick disease, type C1, 257220
NPC2	100%	99,60%	100%	100%	Niemann-pick disease, type C2, 607625
NT5C2	98,00%	96,50%	100%	100%	Spastic paraplegia 45, autosomal recessive, 613162
NUP62	100%	100%	100%	100%	Striatonigral degeneration, infantile, 271930
OCLN	100%	100%	100%	100%	Pseudo-TORCH syndrome 1, 251290
OPA1	99,70%	97,60%	100%	100%	Behr syndrome, 210000<br>Optic atrophy 1, 165500<br>Optic atrophy plus syndrome, 125250<br>?Mitochondrial DNA depletion syndrome 14 (encephalocardiomyopathic type), 616896
OPHN1	99,50%	97,60%	99,90%	98,80%	Mental retardation, X-linked, with cerebellar hypoplasia and distinctive facial appearance, 300486
PACS2	99,30%	96,20%	100%	99,80%	Epileptic encephalopathy, early infantile, 66, 618067
PANK2	100%	99,30%	100%	100%	HARP syndrome, 607236<br>Neurodegeneration with brain iron accumulation 1, 234200
PAX6	100%	100%	100%	100%	Optic nerve hypoplasia, 165550<br>?Coloboma, ocular, 120200<br>Foveal hypoplasia 1, 136520<br>Aniridia, 106210<br>Keratitis, 148190<br>?Coloboma of optic nerve, 120430<br>?Morning glory disc anomaly, 120430<br>Cataract with late-onset corneal dystrophy, 106210<br>Anterior segment dysgenesis 5, multiple subtypes, 604229
PCYT2	99,80%	97,10%	100%	98,80%	Spastic paraplegia 82, autosomal recessive, 618770
PDE10A	81,20%	80,50%	100%	100%	Dyskinesia, limb and orofacial, infantile-onset, 616921<br>Striatal degeneration, autosomal dominant, 616922

PDE8B	99,90%	99,70%	100%	100%	Striatal degeneration, autosomal dominant, 609161<br>Pigmented nodular adrenocortical disease, primary, 3, 614190
PDGFB	100%	99,30%	100%	100%	Dermatofibrosarcoma protuberans, 607907<br>Basal ganglia calcification, idiopathic, 5, 615483<br>Meningioma, SIS-related, 607174
PDGFRB	99,20%	97,50%	100%	100%	Basal ganglia calcification, idiopathic, 4, 615007<br>Kosaki overgrowth syndrome, 616592<br>Myofibromatosis, infantile, 1, 228550<br>Premature aging syndrome, Penttinen type, 601812
PDHA1	99,40%	97,10%	100%	100%	Pyruvate dehydrogenase E1-alpha deficiency, 312170
PDHX	99,90%	99,40%	100%	100%	Lacticacidemia due to PDX1 deficiency, 245349
PDSS1	94,70%	87,60%	97,30%	96,60%	Coenzyme Q10 deficiency, primary, 2, 614651
PDSS2	99,80%	97,10%	100%	100%	Coenzyme Q10 deficiency, primary, 3, 614652
PDYN	100%	100%	100%	100%	Spinocerebellar ataxia 23, 610245
PEX10	96,80%	89,70%	100%	99,90%	Peroxisome biogenesis disorder 6B, 614871<br>Peroxisome biogenesis disorder 6A (Zellweger), 614870
PEX2	100%	100%	100%	100%	Peroxisome biogenesis disorder 5A (Zellweger), 614866<br>Peroxisome biogenesis disorder 5B, 614867
PEX7	87,80%	80,70%	91,30%	91,30%	Peroxisome biogenesis disorder 9B, 614879<br>Rhizomelic chondrodysplasia punctata, type 1, 215100
PHYH	100%	99,60%	100%	100%	Refsum disease, 266500
TAF1	99,80%	97,70%	100%	100%	Dystonia-Parkinsonism, X-linked, 314250<br>Mental retardation, X-linked, syndromic 33, 300966
TANGO2	100%	99,30%	100%	100%	metabolic encephalomyopathic crises, recurrent, with rhabdomyolysis, cardiac arrhythmias, and neurodegeneration, 616878
TBC1D20	94,20%	94,20%	100%	99,90%	Warburg micro syndrome 4, 615663
TBC1D23	99,70%	97,20%	100%	100%	Pontocerebellar hypoplasia, type 11, 617695
PIK3R5	100%	99,90%	100%	100%	Ataxia-oculomotor apraxia 3, 615217
PLA2G6	99,90%	98,30%	100%	100%	Infantile neuroaxonal dystrophy 1, 256600<br>Parkinson disease 14, autosomal recessive, 612953<br>Neurodegeneration with brain iron accumulation 2B, 610217
PLP1	100%	99,20%	100%	100%	Pelizaeus-Merzbacher disease, 312080<br>Spastic paraplegia 2, X-linked, 312920
PMM2	100%	100%	100%	100%	Congenital disorder of glycosylation, type Ia, 212065
PMPCA	97,70%	94,20%	100%	100%	Spinocerebellar ataxia, autosomal recessive 2, 213200
PNKD	100%	99,90%	100%	100%	Paroxysmal nonkinesigenic dyskinesia 1, 118800
PNKP	100%	100%	100%	100%	Ataxia-oculomotor apraxia 4, 616267<br>Microcephaly, seizures, and developmental delay, 613402

PNPLA6	100%	99,70%	100%	100%	Spastic paraplegia 39, autosomal recessive, 612020<br>Boucher-Neuhauser syndrome, 215470<br>Oliver-McFarlane syndrome, 275400<br>?Laurence-Moon syndrome, 245800
POLG	100%	99,30%	100%	100%	Progressive external ophthalmoplegia, autosomal dominant 1, 157640<br>Mitochondrial DNA depletion syndrome 4B (MNGIE type), 613662<br>Mitochondrial recessive ataxia syndrome (includes SANDO and SCAE), 607459<br>Mitochondrial DNA depletion syndrome 4A (Alpers type), 203700<br>Progressive external ophthalmoplegia, autosomal recessive 1, 258450
POLR1C	99,30%	95,50%	90,70%	90,70%	Treacher Collins syndrome 3, 248390<br>Leukodystrophy, hypomyelinating, 11, 616494
POLR3A	100%	99,70%	100%	100%	Leukodystrophy, hypomyelinating, 7, with or without oligodontia and/or hypogonadotropic hypogonadism, 607694<br>Wiedemann-Rautenstrauch syndrome, 264090
POLR3B	99,90%	98,60%	100%	100%	Leukodystrophy, hypomyelinating, 8, with or without oligodontia and/or hypogonadotropic hypogonadism, 614381
PRF1	91,20%	90,80%	100%	100%	Aplastic anemia, 609135<br>Lymphoma, non-Hodgkin, 605027<br>Hemophagocytic lymphohistiocytosis, familial, 2, 603553
PRICKLE1	100%	100%	100%	100%	Epilepsy, progressive myoclonic 1B, 612437
PRKCG	99,90%	98,40%	100%	100%	Spinocerebellar ataxia 14, 605361
PRKRA	100%	99,40%	100%	100%	Dystonia 16, 612067
PRRT2	100%	99,60%	100%	100%	Episodic kinesigenic dyskinesia 1, 128200<br>Seizures, benign familial infantile, 2, 605751<br>Convulsions, familial infantile, with paroxysmal choreoathetosis, 602066
PSAP	100%	100%	100%	100%	Gaucher disease, atypical, 610539<br>Krabbe disease, atypical, 611722<br>Combined SAP deficiency, 611721<br>Metachromatic leukodystrophy due to SAP-b deficiency, 249900
PTS	99,90%	99,10%	100%	100%	Hyperphenylalaninemia, BH4-deficient, A, 261640
PUM1	100%	99,90%	100%	100%	Spinocerebellar ataxia 47, 617931
PYCR2	100%	99,10%	100%	100%	Leukodystrophy, hypomyelinating, 10, 616420
QDPR	100%	99,70%	100%	100%	Hyperphenylalaninemia, BH4-deficient, C, 261630
RAB18	99,50%	97,40%	100%	100%	Warburg micro syndrome 3, 614222
RAB3GAP1	99,40%	98,90%	99,40%	99,40%	Warburg micro syndrome 1, 600118
RAB3GAP2	99,50%	97,00%	100%	100%	Warburg micro syndrome 2, 614225<br>Martsolf syndrome, 212720

RAD51	89,40%	89,40%	89,40%	89,40%	?Fanconi anemia, complementation group R, 617244<br>Mirror movements 2, 614508
RARS	94,20%	91,60%	94,40%	94,30%	Leukodystrophy, hypomyelinating, 9, 616140
RARS2	100%	99,80%	100%	100%	Pontocerebellar hypoplasia, type 6, 611523
REEP1	78,70%	76,10%	100%	100%	Spastic paraplegia 31, autosomal dominant, 610250<br>?Neuronopathy, distal hereditary motor, type VB, 614751
RNASEH2A	100%	100%	100%	100%	Aicardi-Goutieres syndrome 4, 610333
RNASEH2B	96,00%	92,50%	100%	99,80%	Aicardi-Goutieres syndrome 2, 610181
RNASEH2C	100%	99,50%	100%	100%	Aicardi-Goutieres syndrome 3, 610329
RNF170	99,60%	97,60%	100%	100%	Ataxia, sensory, 1, autosomal dominant, 608984
RNF216	99,80%	98,70%	100%	100%	Cerebellar ataxia and hypogonadotropic hypogonadism, 212840
RTN2	100%	99,20%	100%	100%	Spastic paraplegia 12, autosomal dominant, 604805
RUBCN	99,40%	97,50%	100%	100%	?Spinocerebellar ataxia, autosomal recessive 15, 615705
SACS	100%	100%	100%	100%	Spastic ataxia, Charlevoix-Saguenay type, 270550
SAMD9L	100%	100%	100%	100%	Ataxia-pancytopenia syndrome, 159550
SAMHD1	100%	99,60%	100%	100%	?Chilblain lupus 2, 614415<br>Aicardi-Goutieres syndrome 5, 612952
SCN11A	99,80%	98,30%	100%	100%	Episodic pain syndrome, familial, 3, 615552<br>Neuropathy, hereditary sensory and autonomic, type VII, 615548
SCN1A	99,90%	99,50%	100%	100%	Febrile seizures, familial, 3A, 604403<br>Migraine, familial hemiplegic, 3, 609634<br>Epilepsy, generalized, with febrile seizures plus, type 2, 604403<br>Epileptic encephalopathy, early infantile, 6 (Dravet syndrome), 607208
SCN8A	100%	99,80%	100%	100%	Seizures, benign familial infantile, 5, 617080<br>Cognitive impairment with or without cerebellar ataxia, 614306<br>?Myoclonus, familial, 2, 618364<br>Epileptic encephalopathy, early infantile, 13, 614558
SEPSECS	100%	100%	100%	100%	Pontocerebellar hypoplasia type 2D, 613811
SERAC1	99,90%	99,50%	100%	100%	3-methylglutaconic aciduria with deafness, encephalopathy, and Leigh-like syndrome, 614739
SETX	100%	99,80%	100%	100%	Spinocerebellar ataxia, autosomal recessive, with axonal neuropathy 2, 606002<br>Amyotrophic lateral sclerosis 4, juvenile, 602433
SGCE	98,70%	94,00%	95,20%	95,20%	Dystonia-11, myoclonic, 159900
SIL1	99,20%	96,70%	100%	100%	Marinesco-Sjogren syndrome, 248800
SLC12A6	100%	100%	100%	100%	Agenesis of the corpus callosum with peripheral neuropathy, 218000
SLC16A2	99,20%	93,70%	100%	100%	Allan-Herndon-Dudley syndrome, 300523
SLC19A3	100%	99,80%	98,70%	98,70%	Thiamine metabolism dysfunction syndrome 2 (biotin- or thiamine-responsive encephalopathy type 2), 607483
SLC1A3	100%	99,90%	100%	100%	Episodic ataxia, type 6, 612656

SLC20A2	100%	99,20%	100%	100%	Basal ganglia calcification, idiopathic, 1, 213600
SLC25A15	99,80%	98,10%	100%	100%	Hyperornithinemia-hyperammonemia-homocitrullinemia syndrome, 238970
SLC2A1	92,80%	92,80%	100%	100%	Dystonia 9, 601042<br>GLUT1 deficiency syndrome 1, infantile onset, severe, 606777<br>Stomatin-deficient cryohydrocytosis with neurologic defects, 608885<br>GLUT1 deficiency syndrome 2, childhood onset, 612126
SLC30A10	100%	100%	100%	100%	Hypermanganesemia with dystonia 1, 613280
SLC33A1	99,90%	98,90%	100%	100%	Spastic paraplegia 42, autosomal dominant, 612539<br>Congenital cataracts, hearing loss, and neurodegeneration, 614482
SLC39A14	100%	99,40%	93,50%	93,50%	?Hyperostosis cranalis interna, 144755<br>Hypermanganesemia with dystonia 2, 617013
SLC52A2	100%	100%	100%	100%	Brown-Vialetto-Van Laere syndrome 2, 614707
SLC52A3	100%	100%	100%	100%	Brown-Vialetto-Van Laere syndrome 1, 211530<br>?Fazio-Londe disease, 211500
SLC6A3	100%	100%	100%	100%	Parkinsonism-dystonia, infantile, 1, 613135
SLC9A1	100%	100%	100%	100%	?Lichtenstein-Knorr syndrome, 616291
SMPD1	100%	100%	100%	100%	Niemann-Pick disease, type A, 257200<br>Niemann-Pick disease, type B, 607616
SNCA	100%	100%	100%	100%	Dementia, Lewy body, 127750<br>Parkinson disease 1, 168601<br>Parkinson disease 4, 605543
SNORD118	NC	NC	NC	NC	Leukoencephalopathy, brain calcifications, and cysts, 614561
SNX14	99,60%	95,90%	100%	100%	Spinocerebellar ataxia, autosomal recessive 20, 616354
SOX10	99,90%	97,90%	100%	100%	Waardenburg syndrome, type 2E, with or without neurologic involvement, 611584<br>PCWH syndrome, 609136<br>Waardenburg syndrome, type 4C, 613266
SPART	99,70%	96,80%	100%	100%	Troyer syndrome, 275900
SPAST	99,80%	98,70%	100%	100%	Spastic paraplegia 4, autosomal dominant, 182601
SPG11	100%	99,30%	100%	100%	Charcot-Marie-Tooth disease, axonal, type 2X, 616668<br>Spastic paraplegia 11, autosomal recessive, 604360<br>Amyotrophic lateral sclerosis 5, juvenile, 602099
SPG21	99,40%	96,80%	100%	100%	Mast syndrome, 248900
SPG7	94,90%	92,60%	100%	100%	Spastic paraplegia 7, autosomal recessive, 607259
SPR	99,80%	96,30%	100%	100%	Dystonia, dopa-responsive, due to sepiapterin reductase deficiency, 612716
SPTBN2	100%	99,30%	99,90%	99,90%	Spinocerebellar ataxia, autosomal recessive 14, 615386<br>Spinocerebellar ataxia 5, 600224
STUB1	100%	98,70%	100%	100%	Spinocerebellar ataxia, autosomal recessive 16, 615768<br>?Spinocerebellar ataxia 48, 618093

SUMF1	97,50%	90,80%	100%	100%	Multiple sulfatase deficiency, 272200
SUOX	100%	100%	100%	100%	Sulfite oxidase deficiency, 272300
SYNE1	98,30%	98,00%	98,80%	98,80%	Spinocerebellar ataxia, autosomal recessive 8, 610743<br>Arthrogryposis multiplex congenita, myogenic type, 618484<br>Emery-Dreifuss muscular dystrophy 4, autosomal dominant, 612998
TBCD	96,20%	94,40%	100%	100%	Encephalopathy, progressive, early-onset, with brain atrophy and thin corpus callosum, 617193
TDP1	99,90%	99,50%	100%	100%	?Spinocerebellar ataxia, autosomal recessive, with axonal neuropathy 1, 607250
TDP2	100%	99,40%	100%	100%	Spinocerebellar ataxia, autosomal recessive 23, 616949
TECPR2	100%	100%	100%	100%	Spastic paraplegia 49, autosomal recessive, 615031
TENM4	100%	99,60%	100%	100%	Essential tremor, hereditary, 5, 616736
TGM6	99,70%	97,30%	100%	100%	Spinocerebellar ataxia 35, 613908
TH	99,30%	96,10%	100%	100%	Segawa syndrome, recessive, 605407
THAP1	100%	100%	100%	100%	Dystonia 6, torsion, 602629
TIMM8A	98,00%	90,10%	100%	100%	Mohr-Tranebjaerg syndrome, 304700
TMEM106B	99,90%	98,80%	100%	100%	Leukodystrophy, hypomyelinating, 16, 617964
TMEM240	100%	100%	100%	100%	Spinocerebellar ataxia 21, 607454
TMEM67	99,50%	95,00%	100%	99,90%	Meckel syndrome 3, 607361<br>?RHYNS syndrome, 602152<br>Nephronophthisis 11, 613550<br>COACH syndrome, 216360<br>Joubert syndrome 6, 610688
TOE1	100%	100%	100%	100%	Pontocerebellar hypoplasia, type 7, 614969
TOR1A	100%	99,90%	100%	100%	Dystonia-1, torsion, 128100
TPP1	100%	100%	100%	100%	Spinocerebellar ataxia, autosomal recessive 7, 609270<br>Ceroid lipofuscinosis, neuronal, 2, 204500
TREM2	100%	99,80%	100%	100%	Polycystic lipomembranous osteodysplasia with sclerosing leukoencephalopathy 2, 618193
TREX1	100%	100%	100%	100%	Vasculopathy, retinal, with cerebral leukodystrophy, 192315<br>Aicardi-Goutieres syndrome 1, dominant and recessive, 225750<br>Chilblain lupus, 610448
TRPM3	100%	99,50%	100%	100%	No OMIM disease ID
TSEN2	100%	99,60%	100%	100%	Pontocerebellar hypoplasia type 2B, 612389
TSEN54	96,30%	94,30%	99,90%	98,90%	Pontocerebellar hypoplasia type 4, 225753<br>Pontocerebellar hypoplasia type 2A, 277470<br>?Pontocerebellar hypoplasia type 5, 610204
TTBK2	99,80%	97,60%	100%	100%	Spinocerebellar ataxia 11, 604432
TTC19	81,50%	73,80%	100%	99,20%	Mitochondrial complex III deficiency, nuclear type 2, 615157
TTPA	94,70%	87,10%	100%	100%	Ataxia with isolated vitamin E deficiency, 277460

TUBA1A	99,90%	97,00%	100%	100%	Lissencephaly 3, 611603
TUBB	97,30%	93,90%	99,80%	99,80%	Symmetric circumferential skin creases, congenital, 1, 156610<br>Cortical dysplasia, complex, with other brain malformations 6, 615771
TUBB4A	95,90%	94,00%	97,10%	96,00%	Leukodystrophy, hypomyelinating, 6, 612438<br>Dystonia 4, torsion, autosomal dominant, 128101
TUBG1	100%	100%	100%	100%	Cortical dysplasia, complex, with other brain malformations 4, 615412
TWNK	100%	100%	100%	100%	Mitochondrial DNA depletion syndrome 7 (hepatocerebral type), 271245<br>Progressive external ophthalmoplegia with mitochondrial DNA deletions, autosomal dominant 3, 609286<br>Perrault syndrome 5, 616138
TYROBP	100%	100%	100%	100%	Polycystic lipomembranous osteodysplasia with sclerosing leukoencephalopathy 1, 221770
UBAP1	98,80%	93,40%	100%	100%	Spastic paraplegia 80, autosomal dominant, 618418
UBTF	100%	99,40%	100%	100%	Neurodegeneration, childhood-onset, with brain atrophy, 617672
VAMP1	100%	100%	100%	100%	Spastic ataxia 1, autosomal dominant, 108600<br>Myasthenic syndrome, congenital, 25, 618323
VARS2	100%	99,40%	100%	100%	Combined oxidative phosphorylation deficiency 20, 615917
VCP	100%	99,20%	100%	100%	Inclusion body myopathy with early-onset Paget disease and frontotemporal dementia 1, 167320<br>Charcot-Marie-Tooth disease, type 2Y, 616687<br>Amyotrophic lateral sclerosis 14, with or without frontotemporal dementia, 613954
VLDLR	100%	99,80%	100%	100%	Cerebellar hypoplasia and mental retardation with or without quadrupedal locomotion 1, 224050
VPS13A	99,40%	95,60%	100%	100%	Choreoacanthocytosis, 200150
VPS13D	100%	99,70%	100%	100%	Spinocerebellar ataxia, autosomal recessive 4, 607317
VPS16	100%	100%	100%	100%	No OMIM disease ID
VPS37A	91,30%	78,20%	100%	100%	Spastic paraplegia 53, autosomal recessive, 614898
VPS53	91,50%	90,70%	100%	99,30%	Pontocerebellar hypoplasia, type 2E, 615851
VRK1	99,70%	98,50%	100%	100%	Pontocerebellar hypoplasia type 1A, 607596
WASHC5	100%	99,80%	100%	100%	Ritscher-Schinzel syndrome 1, 220210<br>Spastic paraplegia 8, autosomal dominant, 603563
WDR26	99,00%	96,60%	100%	100%	Skraban-Deardorff syndrome, 617616
WDR45	96,40%	89,70%	100%	100%	Neurodegeneration with brain iron accumulation 5, 300894
WDR73	100%	100%	100%	100%	Galloway-Mowat syndrome 1, 251300
WDR81	100%	100%	100%	100%	Hydrocephalus, congenital, 3, with brain anomalies, 617967<br>Cerebellar ataxia, mental retardation, and dysequilibrium syndrome 2, 610185
WWOX	100%	100%	100%	100%	Esophageal squamous cell carcinoma, somatic, 133239<br>Spinocerebellar ataxia, autosomal recessive 12, 614322<br>Epileptic encephalopathy, early infantile, 28, 616211
XK	99,80%	98,10%	100%	100%	McLeod syndrome with or without chronic granulomatous disease, 300842
XPR1	100%	99,90%	100%	100%	Basal ganglia calcification, idiopathic, 6, 616413

XRCC1	100%	98,80%	100%	100%	?Spinocerebellar ataxia, autosomal recessive 26, 617633
ZC4H2	100%	99,00%	100%	100%	Wieacker-Wolff syndrome, 314580<br>Wieacker-Wolff syndrome, female-restricted, 301041
ZFYVE26	100%	99,10%	100%	100%	Spastic paraplegia 15, autosomal recessive, 270700
ZFYVE27	100%	100%	100%	100%	Spastic paraplegia 33, autosomal dominant, 610244
ZNF592	100%	99,60%	100%	100%	No OMIM disease ID

Gene symbols used follow HGCN guidelines: Gray KA, Yates B, Seal RL, Wright MW, Bruford EA. Nucleic Acids Res. 2015 Jan 43(Database issue):D1079-85.
Agilent V5 is the default chemistry, and used for all exome analyses apart from the (in-house) TURBO/RAPID WES route.
TWIST is the chemistry used for (in-house) TURBO/RAPID WES analysis.
Covered 10x describes the percentage of a gene's coding sequence that is covered at least 10x.
Covered 20x describes the percentage of a gene's coding sequence that is covered at least 20x.
Genes with coverage denoting NC are non-DNA coding genes.
non-DNA coding genes are covered, but as coverage statistics are based on DNA coding regions, statistics could not be generated.
OMIM release used for OMIM disease identifiers and descriptions : April 20th , 2020.
This list is accurate for panel version DG 2.18
Ad 1. "No OMIM Disease ID" signifies a gene without a current OMIM association Ad 2. OMIM phenotype descriptions between {} signify risk factors
