# Supplementary material for: Genetic characterization of primary lateral sclerosis
Source: J Neurol. 2023 May 3;270(8):3970–80. doi: 10.1007/s00415-023-11746-7 (PMC10345048; doi:10.1007/s00415-023-11746-7)
Supplement: Supplementary file 2 — Supplementary file2 (PDF 111 KB) [file 415_2023_11746_MOESM2_ESM.pdf]

ALS GENE PANEL DG 2.18 (22 genes)

Releasedate: 20-04-2020

Gene	Agilent V5 covered >10x	Agilent V5 covered > 20x	TWIST covered >10x	TWIST covered >20x	Associated Phenotype description and OMIM disease ID
ALS2	100,00%	99,90%	100,00%	100,00%	Primary lateral sclerosis, juvenile, 606353<br>Amyotrophic lateral sclerosis 2, juvenile, 205100<br>Spastic paralysis, infantile onset ascending, 607225
ANG	100,00%	100,00%	100,00%	100,00%	Amyotrophic lateral sclerosis 9, 611895
ANXA11	100,00%	98,50%	100,00%	100,00%	Amytrophic lateral sclerosis 23, 617839
CHCHD10	59,10%	43,90%	100,00%	100,00%	Spinal muscular atrophy, Jokela type, 615048<br>Frontotemporal dementia and/or amyotrophic lateral sclerosis 2, 615911<br>?Myopathy, isolated mitochondrial, autosomal dominant, 616209
CHMP2B	99,70%	96,70%	100,00%	100,00%	Amyotrophic lateral sclerosis 17, 614696<br>Dementia, familial, nonspecific, 600795
ERBB4	100,00%	99,50%	100,00%	100,00%	Amyotrophic lateral sclerosis 19, 615515
FIG4	100,00%	99,80%	100,00%	100,00%	Yunis-Varon syndrome, 216340<br>?Polymicrogyria, bilateral temporooccipital, 612691<br>Charcot-Marie-Tooth disease, type 4J, 611228<br>Amyotrophic lateral sclerosis 11, 612577
FUS	99,20%	96,40%	100,00%	100,00%	Amyotrophic lateral sclerosis 6, with or without frontotemporal dementia, 608030<br>Essential tremor, hereditary, 4, 614782
MATR3	97,00%	93,40%	100,00%	100,00%	Amyotrophic lateral sclerosis 21, 606070
OPTN	100,00%	99,90%	100,00%	100,00%	Glaucoma 1, open angle, E, 137760<br>Amyotrophic lateral sclerosis 12, 613435
PFN1	100,00%	100,00%	100,00%	100,00%	Amyotrophic lateral sclerosis 18, 614808
TARDBP	100,00%	100,00%	100,00%	100,00%	Frontotemporal lobar degeneration, TARDBP-related, 612069<br>Amyotrophic lateral sclerosis 10, with or without FTD, 612069
SETX	100,00%	99,80%	100,00%	100,00%	Spinocerebellar ataxia, autosomal recessive, with axonal neuropathy 2, 606002<br>Amyotrophic lateral sclerosis 4, juvenile, 602433
SIGMAR1	100,00%	100,00%	100,00%	100,00%	?Amyotrophic lateral sclerosis 16, juvenile, 614373<br>?Spinal muscular atrophy, distal, autosomal recessive, 2, 605726
SOD1	100,00%	99,90%	100,00%	100,00%	Amyotrophic lateral sclerosis 1, 105400<br>Spastic tetraplegia and axial hypotonia, progressive, 618598

SPG11	100,00%	99,30%	100,00%	100,00%	Charcot-Marie-Tooth disease, axonal, type 2X, 616668<br>Spastic paraplegia 11, autosomal recessive, 604360<br>Amyotrophic lateral sclerosis 5, juvenile, 602099
SQSTM1	98,80%	95,50%	100,00%	100,00%	Frontotemporal dementia and/or amyotrophic lateral sclerosis 3, 616437<br>Neurodegeneration with ataxia, dystonia, and gaze palsy, childhood-onset, 617145<br>Myopathy, distal, with rimmed vacuoles, 617158<br>Paget disease of bone 3, 167250
TBK1	99,70%	97,20%	100,00%	100,00%	Frontotemporal dementia and/or amyotrophic lateral sclerosis 4, 616439
TUBA4A	100,00%	100,00%	100,00%	100,00%	Amyotrophic lateral sclerosis 22 with or without frontotemporal dementia, 616208
UBQLN2	100,00%	99,40%	100,00%	100,00%	Amyotrophic lateral sclerosis 15, with or without frontotemporal dementia, 300857
VAPB	100,00%	99,90%	100,00%	100,00%	Spinal muscular atrophy, late-onset, Finkel type, 182980<br>Amyotrophic lateral sclerosis 8, 608627
VCP	100,00%	99,20%	100,00%	100,00%	Inclusion body myopathy with early-onset Paget disease and frontotemporal dementia 1, 167320<br>Charcot-Marie-Tooth disease, type 2Y, 616687<br>Amyotrophic lateral sclerosis 14, with or without frontotemporal dementia, 613954

Gene symbols used follow HGCN guidelines: Gray KA, Yates B, Seal RL, Wright MW, Bruford EA. Nucleic Acids Res. 2015 Jan 43(Database issue):D1079-85.
Agilent V5 is the default chemistry, and used for all exome analyses apart from the (in-house) TURBO/RAPID WES route.
TWIST is the chemistry used for (in-house) TURBO/RAPID WES analysis.
Covered 10x describes the percentage of a gene's coding sequence that is covered at least 10x.
Covered 20x describes the percentage of a gene's coding sequence that is covered at least 20x.
Genes with coverage denoting NC are non-DNA coding genes.
non-DNA coding genes are covered, but as coverage statistics are based on DNA coding regions, statistics could not be generated.
OMIM release used for OMIM disease identifiers and descriptions : April 20th , 2020.
This list is accurate for panel version DG 2.18
Ad 1. "No OMIM Disease ID" signifies a gene without a current OMIM association Ad 2. OMIM phenotype descriptions between {} signify risk factors
